# A scoping review protocol on diagnostic strategies to detect occult malignancies in individuals with ischemic stroke

**DOI:** 10.1371/journal.pone.0289048

**Published:** 2023-07-21

**Authors:** Jenneke Leentjens, Nicholas L. J. Chornenki, Janneke Spiegelenberg, Valentina Ly, Dariush Dowlatshahi, Deborah M. Siegal

**Affiliations:** 1 Department of Internal Medicine & Radboud Institute for Health Sciences, Radboud University Medical Center, Nijmegen, The Netherlands; 2 Internal Medicine Residency Program, Queens University, Kingston, Canada; 3 Synapse Research Institute, Maastricht, The Netherlands; 4 Health Sciences Library, University of Ottawa, Ottawa, Canada; 5 Department of Medicine, University of Ottawa, Ottawa, Canada; Hamad Medical Corporation, QATAR

## Abstract

**Background:**

Emerging data show an increased risk of ischemic stroke in patients with a new diagnosis of cancer. As the risk of stroke begins to increase 150 days before cancer is diagnosed, stroke may be the first clinical manifestation of undiagnosed cancer. About 6% of patients with cryptogenic ischemic stroke (unknown etiology after diagnostic evaluations) are diagnosed with cancer within one year. However, the optimal cancer screening strategy in this population is not known. We aim to conduct a scoping review of screening strategies for occult cancer in individuals with ischemic stroke.

**Methods:**

Electronic databases including MEDLINE (Ovid), EMBASE (Ovid), CINAHL (EBSCOhost) and Scopus will be systematically searched to identify articles that report on screening strategies for occult cancer in individuals with ischemic stroke. At least two investigators will independently perform two-stage study selection consisting of title/abstract screening and full-text review, followed by data extraction. Thereafter, a thematic analysis will be conducted to provide an overview of what diagnostic tests/strategies have been used, and their clinical utility in terms of positive and negative predictive value (when available).

**Conclusion:**

We anticipate that the findings of this scoping review will identify strategies used to detect occult cancer in individuals with ischemic stroke and summarize their clinical utility (if reported). Addressing this knowledge gap will help guide the development of future clinical trials on occult cancer screening patients with ischemic stroke.

## Introduction

Ischemic stroke is a leading cause of disability and mortality worldwide, affecting millions of people [1, 2]. Determining stroke etiology is key for optimal secondary stroke prevention, but remains unidentified in 10% to 40% of patients even after modern diagnostic workup (so-called cryptogenic stroke) [3, 4]. The current definition of cryptogenic stroke is based on the definition used in the trial of ORG 10172 in Acute Stroke Treatment (TOAST) [5]. Cryptogenic stroke (or “stroke of undetermined etiology” in TOAST terminology) is defined as an ischemic brain infarction not attributable to large-artery atherosclerosis, small artery disease, or cardioembolism despite a standard vascular, cardiac, and serologic evaluation (Table 1). Emerging data suggest that active cancer is an important underlying cause of ischemic stroke in individuals with cryptogenic stroke, with unique pathophysiology and treatment considerations [6]. Several large studies have demonstrated an increased risk of ischemic stroke in patients with new cancer diagnoses compared to matched controls [7, 8]. It is also increasingly recognized that stroke can precede cancer diagnosis by several months indicating that stroke may be the first manifestation of underlying cancer [9, 10]. Up to 10% of individuals with cryptogenic ischemic stroke are diagnosed with cancer within one year, which is similar to the incidence of occult cancer in individuals with unprovoked (no identifiable major risk factors) thromboembolism (VTE) [10–12]. However, compared to the extensive literature on cancer associated VTE, research on cancer associated stroke is more limited [11]. Although experts endorse consideration of underlying occult cancer as an etiology of cryptogenic stroke [6, 13, 14], specific recommendations are lacking and no advice is given in current guidelines [15–20]. Given the uncertainty about the extent and type of existing literature regarding screening for occult cancer in patients with ischemic stroke, we will map the available evidence in this scoping review in order to identify current knowledge gaps prior to undertaking a systematic review.

**Table 1:** Routine workup according to TOAST criteria for stroke of undetermined etiology [13].

Brain imaging by CT or MRI
Duplex ultrasound of the neck and transcranial doppler of the intracranial arteries or CT angiography of MR angiography of the neck and head arteries
Extensive cardiac workup (standard 12-lead electrocardiogram (ECG), at least 24-hour cardiac monitoring after stroke onset to detect subclinical atrial fibrillation, and transthoracic echocardiography)
Routine blood tests include blood glucose levels, complete blood count, International normalized ratio (INR), prothrombin time, activated partial thromboplastin time, and blood lipids

## Methods

This scoping review will be conducted according to the methodology proposed by Arksey and O’Malley [21, 22] which consists of nine stages as follows: 1) identify the research question(s); 2) develop the inclusion criteria; 3) describe the planned approach to evidence searching, selection, data extraction, and presentation; 4) search for the evidence; 5) select the evidence; 6) extract the evidence; 7) analyze the evidence; 8) present the results; 9) summarize the evidence in relation to the purpose of the review, making conclusions and noting any implications of the findings. This study is registered with the Open Science Framework (https://osf.io/3h95q). The results will be reported according to the Preferred Reporting Items for Systematic reviews and Meta-Analyses extension for Scoping Reviews (PRISMA-ScR).

### Research question

In patients with cryptogenic ischemic stroke, have occult cancer screening strategies been evaluated and, if so, what tests have been used and what is the clinical value in terms of positive and negative predictive value?

### Inclusion and exclusion criteria

All articles that describe strategies for detecting occult cancers in adult patients with ischemic stroke (including, but not limited to, imaging tests, laboratory investigations, clinical assessments) will be assessed. Eligible articles include expert opinions, guidelines, narrative and systematic reviews, case-series and case-reports, conference abstracts, observational and randomized studies. Non-English reports, animal studies, studies limited to intracerebral hemorrhage, studies in patients with known cancers, studies on cardioembolic ischemic stroke (including fibroelastoma, atrial myxoma, and non-bacterial thrombotic endocarditis), and studies without details about screening strategies will be excluded. The reference lists of articles selected for full-text review will also be reviewed for potentially eligible studies that were not captured by the search.

### Search strategy and information sources

The search strategy was developed by a health sciences librarian (VL) and peer-reviewed by an independent health sciences librarian from the University of Ottawa (Tables 2–5) [23]. MEDLINE (Ovid), Embase (Ovid), CINAHL (EBSCO), and Scopus were searched with no limits to language or publication date using appropriate subject headings and keywords (see S1 Checklist for full search details). The main search concepts comprise of terms related to ischemic stroke and cancer screening. Search results were exported to Covidence (Melbourne, Australia) and duplicates were eliminated using the platform’s duplicate identification feature.

**Table 2:** Medline (Ovid) search strategy.

#	Searches	Results
1	neoplasms/ and (screen* or test* or diagnos* or detect* or predict* or identif* or scan* or biomarker* or marker* or metabolite* or biops*).ti,ab,kf.	169280
2	early detection of cancer/	34210
3	biomarkers, tumor/	172208
4	((oncolog* or cancer* or carcinoma* or tumor* or tumour* or neoplasm* or metasta* or malignan* or hodgkin* or nonhodgkin* or adenocarcinoma* or leukemi* or leukaemi* or lymphoma* or sarcoma* or myeloma* or melanoma*) adj5 (screen* or test* or diagnos* or detect* or predict* or identif* or scan* or biomarker* or marker* or metabolite* or biops*)).ti,ab,kf.	872000
5	or/1-4	1049172
6	exp ischemic stroke/	7015
7	stroke/ and (ischemi* or ischaemi* or cryptogenic* or wake* or awak* or embol* or cardioembol* or thrombo* or lacunar).ti,ab,kf.	57085
8	((ischemi* or ischaemi* or cryptogenic* or wake* or awak* or embol* or cardioembol* or thrombo*) adj3 (stroke* or cerebr* vascul* or cerebrovascul* or CVA or CVAs or apoplex*)).ti,ab,kf.	90997
9	((ischemi* or ischaemi* or cryptogenic* or wake* or awak* or embol* or cardioembol* or thrombo*) adj3 (brain adj2 (insult* or accident* or attack*))).ti,ab,kf.	249
10	(lacunar adj2 (stroke* or syndrome* or infarct*)).ti,ab,kf.	4133
11	or/6-10	106634
12	5 and 11	930

**Table 3:** Embase (Ovid) search strategy.

#	Searches	Results
1	neoplasm/ and (screen* or test* or diagnos* or detect* or predict* or identif* or scan* or biomarker* or marker* or metabolite* or biops*).ti,ab,kf.	287402
2	cancer screening/	89245
3	tumor marker/	93722
4	((oncolog* or cancer* or carcinoma* or tumor* or tumour* or neoplasm* or metasta* or malignan* or hodgkin* or nonhodgkin* or adenocarcinoma* or leukemi* or leukaemi* or lymphoma* or sarcoma* or myeloma* or melanoma*) adj5 (screen* or test* or diagnos* or detect* or predict* or identif* or scan* or biomarker* or marker* or metabolite* or biops*)).ti,ab,kf.	1317283
5	or/1-4	1533724
6	exp ischemic stroke/	14021
7	cerebrovascular accident/ and (ischemi* or ischaemi* or cryptogenic* or wake* or awak* or embol* or cardioembol* or thrombo* or lacunar).ti,ab,kf.	90841
8	((ischemi* or ischaemi* or cryptogenic* or wake* or awak* or embol* or cardioembol* or thrombo*) adj3 (stroke* or cerebr* vascul* or cerebrovascul* or CVA or CVAs or apoplex*)).ti,ab,kf.	153077
9	((ischemi* or ischaemi* or cryptogenic* or wake* or awak* or embol* or cardioembol* or thrombo*) adj3 (brain adj2 (insult* or accident* or attack*))).ti,ab,kf.	373
10	(lacunar adj2 (stroke* or syndrome* or infarct*)).ti,ab,kf.	6973
11	or/6-10	195402
12	5 and 11	2772

**Table 4:** CINAHL (EBSCO) search strategy.

#	Searches	Results
S1	(MH "Neoplasms")	89,751
S2	TI ((screen* or test* or diagnos* or detect* or predict* or identif* or scan* or biomarker* or marker* or metabolite* or biops*)) OR AB ((screen* or test* or diagnos* or detect* or predict* or identif* or scan* or biomarker* or marker* or metabolite* or biops*))	2,239,282
S3	S1 AND S2	33,284
S4	(MH "Early Detection of Cancer")	11,657
S5	(MH "Tumor Markers, Biological")	16,863
S6	TI (((oncolog* or cancer* or carcinoma* or tumor* or tumour* or neoplasm* or metasta* or malignan* or hodgkin* or nonhodgkin* or adenocarcinoma* or leukemi* or leukaemi* or lymphoma* or sarcoma* or myeloma* or melanoma*) N5 (screen* or test* or diagnos* or detect* or predict* or identif* or scan* or biomarker* or marker* or metabolite* or biops*))) OR AB (((oncolog* or cancer* or carcinoma* or tumor* or tumour* or neoplasm* or metasta* or malignan* or hodgkin* or nonhodgkin* or adenocarcinoma* or leukemi* or leukaemi* or lymphoma* or sarcoma* or myeloma* or melanoma*) N5 (screen* or test* or diagnos* or detect* or predict* or identif* or scan* or biomarker* or marker* or metabolite* or biops*)))	177,839
S7	S3 OR S4 OR S5 OR S6	204,995
S8	(MH "Ischemic Stroke+")	642
S9	(MH "Stroke")	76,233
S10	TI ((ischemi* or ischaemi* or cryptogenic* or wake* or awak* or embol* or cardioembol* or thrombo* or lacunar)) OR AB ((ischemi* or ischaemi* or cryptogenic* or wake* or awak* or embol* or cardioembol* or thrombo* or lacunar))	178,863
S11	S9 AND S10	22,165
S12	TI (((ischemi* or ischaemi* or cryptogenic* or wake* or awak* or embol* or cardioembol* or thrombo*) N3 (stroke* or "cerebr* vascul*" or cerebrovascul* or CVA or CVAs or apoplex*))) OR AB (((ischemi* or ischaemi* or cryptogenic* or wake* or awak* or embol* or cardioembol* or thrombo*) N3 (stroke* or "cerebr* vascul*" or cerebrovascul* or CVA or CVAs or apoplex*)))	30,904
S13	TI (((ischemi* or ischaemi* or cryptogenic* or wake* or awak* or embol* or cardioembol* or thrombo*) N3 (brain N2 (insult* or accident* or attack*)))) OR AB (((ischemi* or ischaemi* or cryptogenic* or wake* or awak* or embol* or cardioembol* or thrombo*) N3 (brain N2 (insult* or accident* or attack*))))	63
S14	(MH "Stroke, Lacunar")	293
S15	TI ((lacunar N2 (stroke* or syndrome* or infarct*))) OR AB ((lacunar N2 (stroke* or syndrome* or infarct*)))	1,286
S16	S8 OR S11 OR S12 OR S13 OR S14 OR S15	35,765
S17	S7 AND S16	318

**Table 5:** Scopus search strategy.

#	Searches	Results
1	TITLE-ABS-KEY ((oncolog* OR cancer* OR carcinoma* OR tumor* OR tumour* OR neoplasm* OR metasta* OR malignan* OR hodgkin* OR nonhodgkin* OR adenocarcinoma* OR leukemi* OR leukaemi* OR lymphoma* OR sarcoma* OR myeloma* OR melanoma*) W/5 (screen* OR test* OR diagnos* OR detect* OR predict* OR identif* OR scan* OR biomarker* OR marker* OR metabolite* OR biops*))	1.324.376
2	TITLE-ABS-KEY ((ischemi* OR ischaemi* OR cryptogenic* OR wake* OR awak* OR embol* OR cardioembol* OR thrombo*) W/3 (stroke* OR "cerebr* vascul*" OR cerebrovascul* OR cva OR cvas OR apoplex*))	108.780
3	TITLE-ABS-KEY ((ischemi* OR ischaemi* OR cryptogenic* OR wake* OR awak* OR embol* OR cardioembol* OR thrombo*) W/3 (brain W/2 (insult* OR accident* OR attack*)))	848
4	TITLE-ABS-KEY (lacunar W/2 (stroke* OR syndrome* OR infarct*))	6.316
5	(TITLE-ABS-KEY ((ischemi* OR ischaemi* OR cryptogenic* OR wake* OR awak* OR embol* OR cardioembol* OR thrombo*) W/3 (stroke* OR "cerebr* vascul*" OR cerebrovascul* OR cva OR cvas OR apoplex*))) OR (TITLE-ABS-KEY ((ischemi* OR ischaemi* OR cryptogenic* OR wake* OR awak* OR embol* OR cardioembol* OR thrombo*) W/3 (brain W/2 (insult* OR accident* OR attack*)))) OR (TITLE-ABS-KEY (lacunar W/2 (stroke* OR syndrome* OR infarct*)))	113.600
6	(TITLE-ABS-KEY ((oncolog* OR cancer* OR carcinoma* OR tumor* OR tumour* OR neoplasm* OR metasta* OR malignan* OR hodgkin* OR nonhodgkin* OR adenocarcinoma* OR leukemi* OR leukaemi* OR lymphoma* OR sarcoma* OR myeloma* OR melanoma*) W/5 (screen* OR test* OR diagnos* OR detect* OR predict* OR identif* OR scan* OR biomarker* OR marker* OR metabolite* OR biops*))) AND ((TITLE-ABS-KEY ((ischemi* OR ischaemi* OR cryptogenic* OR wake* OR awak* OR embol* OR cardioembol* OR thrombo*) W/3 (stroke* OR "cerebr* vascul*" OR cerebrovascul* OR cva OR cvas OR apoplex*))) OR (TITLE-ABS-KEY ((ischemi* OR ischaemi* OR cryptogenic* OR wake* OR awak* OR embol* OR cardioembol* OR thrombo*) W/3 (brain W/2 (insult* OR accident* OR attack*)))) OR (TITLE-ABS-KEY (lacunar W/2 (stroke* OR syndrome* OR infarct*))))	979

### Article selection

We will use a two-stage study selection of title and abstract screening, followed by full-text review, to be completed by two independent reviewers [24]. Disagreements will be resolved by discussion, or involvement of a third reviewer if consensus cannot be reached. A PRISMA flow diagram will be used to summarize the process of article selection.

### Data extraction and management

For studies that meet the inclusion criteria, relevant data will be extracted and captured using a standardized data extraction form. Specific data to be collected are shown in Table 6.

**Table 6:** Data included in the standardized data extraction form.

First author(s)
Year of publication
Origin/country of origin (where the study was published or conducted)
Objective(s)Study design
Duration of follow-up*
Study population (inclusion and exclusion criteria)*
Sample size*
Intervention type/duration, comparator*
Outcome measures * • Definition of cancer diagnosis
Key findings: screening strategies/diagnostic tests used (including but not limited to laboratory tests, imaging investigations, clinical characteristics), PPV/NPV*

*if applicable.

## Conclusion

The objective of the scoping review described in this protocol is to summarize the available evidence on screening strategies to detect occult cancer in individuals with ischemic stroke. We anticipate that this review will summarize the type of tests and/or strategies that are used in clinical practice and provide preliminary evidence about their utility, but there will be heterogeneity due to existing knowledge gaps about the optimal strategy. Information gathered from this review will also help to guide the development of future clinical trials on screening strategies for occult cancer in individuals with ischemic stroke.

## Supporting information

S1 Checklist PRISMA-P (Preferred Reporting Items for Systematic review and Meta-Analysis Protocols) 2015 checklist: Recommended items to address in a systematic review protocol*. (DOC)
