# Supplementary material for: A scoping review protocol on diagnostic strategies to detect occult malignancies in individuals with ischemic stroke
Source: PLoS One. 2023 Jul 21;18(7):e0289048. doi: 10.1371/journal.pone.0289048 (PMC10361530; doi:10.1371/journal.pone.0289048)
Supplement: Attachment — Submitted filename: peerrevew_PLOS.docx [file pone.0289048.s002.docx]

Dear editor,

I reviewed the article received by Leentjens et all on scoping review on cancer as possible etiology of infarctive stroke. The protocol is well written and the strategies part in the tables in elucidated well.

I have just one question for the authors

1. Have figured out any statistical analysis plans for the data? If yes what kind of data would the team extract and how are they planning on analysing it?
2. While its clearly a scoping review, I am interested to know how have the authors planned on presenting the findings from expert opinions, narratives and guidelines which they listed as part of inclusion criteria. Will they present descriptively side by side with data analysis?

If there is a plan to run statistical analysis, the authors need to elaborate on that clearly. Otherwisethere is any need to update the manuscript.

I wish the authors best of luck with their submission and the review
