# Supplementary material for: A scoping review protocol on diagnostic strategies to detect occult malignancies in individuals with ischemic stroke
Source: PLoS One. 2023 Jul 21;18(7):e0289048. doi: 10.1371/journal.pone.0289048 (PMC10361530; doi:10.1371/journal.pone.0289048)
Supplement: Attachment — Submitted filename: Response to reviewer_scoping review protocol def.docx [file pone.0289048.s003.docx]

Dear Dr Fateen Ata,

Thank you very much for the evaluation of our manuscript. Below, we have provided a point-to-point response to the suggestions of the reviewers and we indicated the changes made to the manuscript. In addition, we have provided a marked up version using “ track changes” to indicate the changes made, as well as a clean version using only black font

Yours sincerely,

On behalf of the co-authors,

Jenneke Leentjens

Dear editor,

I reviewed the article received by Leentjens et all on scoping review on cancer as possible etiology of infarctive stroke. The protocol is well written and the strategies part in the tables in elucidated well.

I have just one question for the authors

1. Have figured out any statistical analysis plans for the data? If yes what kind of data would the team extract and how are they planning on analysing it?

***R: The objective of our review is to summarize the evidence from available studies .*** ***We will use descriptive statistics to summarize our findings including mean with standard deviation, or median with interquartile range for continuous variables, and proportions for categorical variables, where appropriate. We will calculate the positive/negative predictive value of a diagnostic test, when available but will not perform comparative analyses between studies, nor will we pool the results in meta-analysis.***

2. While it is clearly a scoping review, I am interested to know how have the authors planned on presenting the findings from expert opinions, narratives and guidelines which they listed as part of inclusion criteria. Will they present descriptively side by side with data analysis?

***R: We will summarize the recommendations from narrative reviews and guidelines in separately from other studies. The type of article (narrative review/guideline) will be stated in the “study design” section and their recommendations in the “key-findings” section. For narrative reviews/guidelines all other items in the chart will be scored as “not applicable”.***

3. If there is a plan to run statistical analysis, the authors need to elaborate on that clearly. Otherwise there is no need to update the manuscript.

***R: we do not plan to run perform comparative analysis nor will we pool the results in meta-analysis.***

I wish the authors best of luck with their submission and the review
